# Ischemic colitis induced by psychotropics drugs: a case report

**DOI:** 10.1192/j.eurpsy.2022.1830

**Published:** 2022-09-01

**Authors:** C. Alvarez Garcia, A. Sanz Giancola, L. Nocete Navarro

**Affiliations:** Hospital Universitario Príncipe de Asturias, Psychiatry, Alcalá de Henares, Spain

**Keywords:** Ischemic colitis, side effect, anticholinergic, psychopharmaceuticals

## Abstract

**Introduction:**

Ischemic colitis (IC) is a rare condition due to hypoperfusion in the large intestine. Usually the etiology is unidentified, but many drugs are known to induce it because of their anticholinergic effects. We present the case of a 63-year-old woman, with the diagnosis of histrionic personality disorder, in treatment with quetiapine and venlafaxine. She attended the hospital due to diffuse abdominal pain, diarrhea and hematochezia in the last two days. An abdominal CT scan is made, showing parietal thickening and submucosal edema in the colon, without any tumoral findings, suggesting IC.

**Objectives:**

To point up the correlation between IC and the intake of psychotropic drugs.

**Methods:**

We conducted a narrative review of the literature through the presentation of a case. Articles were selected based on their clinical relevance.

**Results:**

There are reported cases of IC related to antipsychotics, but any drug with anticholinergic effects can potentially cause it. Anticholinergics reduce intestinal motility, leading to colonic ileus and dilatation. Both quetiapine and venlafaxine, taken by the patient, have these effects. Common obstructive and non-obstructive processes are excluded due to the absence of any other pathological signs. For these reasons, the diagnosis of IC secondary to treatment with quetiapine and venlafaxine is made.

**Conclusions:**

Many psychotropic drugs can produce IC owing to their anticholinergic effects, being this chance increased when taken simultaneously with other drugs with same effects. IC is a rare but fatal side effect, which makes it important to consider it in the differential diagnosis in patients in treatment with psychotropics who suffer from gastrointestinal symptoms.

**Disclosure:**

No significant relationships.

